# Essential oil-incorporated carbon nanotubes filters for bacterial removal and inactivation

**DOI:** 10.1371/journal.pone.0227220

**Published:** 2019-12-27

**Authors:** Xiuli Dong, Ambrose E. Bond, Liju Yang

**Affiliations:** Department of Pharmaceutical Sciences and Biomanufacturing Research Institute and Technology Enterprise (BRITE), North Carolina Central University, Durham, NC, United States of America; VIT University, INDIA

## Abstract

In this study, essential oils (EO)-incorporated multi-walled carbon nanotubes (MWCNTs) filters were developed for achieving dual functions in effective removing bacteria from aqueous solutions and inactivating bacteria cells captured on the filters. Tea tree essential oil (TTO), lemon essential oil (LEO), and TTO-LEO-mixture were coated on MWCNTs filters with different MWCNTs loadings ranging from 3 mg to 6 mg. MWCNTs filters with 6.0 mg MWCNTs showed complete removal (100%) of *E*. *coli* cells from PBS buffer with 6.35 log10 decrease of cell numbers. TTO, LEO, and TTO/LEO Mix (1:1) coatings at the volume of 50 μL on MWCNTs filters achieved bacterial removal rates of >98%, and highly effective inactivation efficiency. TTO coatings had the highest antimicrobial efficacies than LEO and Mix coatings, MWCNTs filters with 50 μL TTO coating showed 100% inhibitory rate of the captured bacteria on the filter surfaces. Those captured but survived cells on filters with less TTO coating (20μL) significantly reduced their salt tolerances to 30 and 40 g/L NaCl in LB agar, and became less salt tolerance with longer incubation time on the filters. The developed TTO-MWCNTs filters had much higher antimicrobial efficacies than the filters with dual functions developed previously.

## Introduction

Waterborne diseases caused by pathogens account for 3.4 million deaths each year [1]. The occurrence of pathogenic microorganisms in contaminated water is a serious issue for almost all types of ambient water bodies. The United Nations identified improvement of water quality as one of the eight Millennium Development Goals (MDGs) in 2000. It aimed to reduce the number of people without the access to safe water by 50% by 2015 and this goal was achieved. However, there are still 663 million people using unimproved drinking water source [2]. To protect public health in the United States, the U.S. Environmental Protection Agency (EPA) has established the National Primary Drinking Water Regulations which set the standards of maximum contaminant level (MCL) of pathogens and require routine sampling of drinking water for testing total coliforms and *E*. *coli* [1, 3].

For eliminating pathogenic microorganisms from water, chemical inactivation or physical removal methods are commonly used. The chemicals used to inactivate microorganisms include gaseous chlorine (Cl_2_), liquid sodium hypochlorite, chloramine, chlorine dioxide, chloramines, hydrogen peroxide, bromine, etc. [4]. Although the application of these chemicals has decreased the number of waterborne diseases dramatically, their potential health risk for drinking water has gained a lot of attention. For example, chlorine, chlorite, and chlorate were found to be able to alter DNA and damage kidneys. Chlorine disinfectant by-products are found in drinking water, including chloroform, bromodichloromethane (BDCM), dibromoacetic acid (DBA), dichloroacetic acid (DCA), trichloroacetic acid (TCA), etc. These by-products were found to be renal carcinogenic and/or showed tumor promoting activities in mice [5]. Chloroform as one of the by-products in chlorinated water is known to be carcinogenic and causes damage to liver, which can further develop to cancer. Other studies reported that exposure to disinfection by-products was associated with induction of bladder and colon cancers and adverse pregnancy outcomes [6–8]. On the other hand, physical removal of microorganisms from contaminated water by using filters is a safer practice which can avoid the health issues caused by the exposure to the harmful disinfectants and their byproducts. However, the efficiency of traditional membrane microfiltration for bacteria removal is very limited, only about 61% [9].

Recent studies reported that using nanofibrous materials could effectively improve filtration systems due to their high permeability and small pore size, demonstrating the potential of nanomaterials for the development of more efficient and cost-effective water filtration processes compared to conventional membrane filtration technologies [10]. Carbon nanotubes (CNTs) are a class of nanomaterials that have been explored for building filters for the removal of contaminant species, desalination, and water purification [11], due to their advantageous features such as fast water transport, large surface area, ease of functionalization, and high adsorption capacity [4]. For example, CNTs can be used as size exclusion membrane filters that allow the flow of water while adsorbing and blocking the flow of contaminants [12]. Our previous studies also demonstrated that membrane filters coated with multi-walled carbon nanotubes (MWCNTs) could effectively capture and remove bacteria from water [4, 13].

Further inactivation of captured microorganisms on the filters would be an additional advantage for the filters. Our previous studies demonstrated the coupling of antimicrobial elements to CNT-coated filters is a feasible strategy to achieve this goal. For example, additional antimicrobial coatings of natural peptide nisin [13], carbon dots (CDots) [4], or single walled carbon nanotubes (SWCNTs) [4] onto MWCNTs filters could inactivate ~73–97% of bacterial cells captured on the filters [4, 13].

This study aimed to achieve completely (100%) removal of bacteria in aqueous samples and to completely inactivate captured bacteria on the filters by coupling more effective non-toxic antimicrobial elements on MWCNTs filters. Among the non-toxic natural antimicrobial agents, essential oils (EOs) could be a good choice as an effective coating to afford filters with antimicrobial function, since they are safe and beneficial to the health of human beings. EOs have been used in aromatherapy and for the treatment of cardiovascular disease, diabetes, Alzheimer’s, and cancer.[14]. EOs are also widely used in the cosmetic, food, perfume, and pharmaceutical industries, with many of them showing antimicrobial effects. Mechanistically, the components in EO, such as different types of aldehydes, phenolics, terpenes, and other compounds, contribute to their highly effective antimicrobial properties against a diverse range of pathogens [14]. For example, EOs derived from *Cymbopogan citratus* had their minimum inhibitory concentrations (MICs) of 0.6, 2.5, and 0.6 μL/mL against *E*. *coli*, *S*. *typhimurim*, and *S*.*aureus* bacteria, respectively, which are the lowest MICs among the chemicals for preventing visible growth of bacterium [15]. Due to their low to no toxicity, EOs have been used on antimicrobial surface coatings, for enhancing the antioxidant, antibacterial, and antifungal efficacy of different types of surfaces, including food industry applications. For example, EO-incorporated films and coatings showed greater effectiveness against foodborne bacteria and postharvest fungi in food systems than pure films and coatings [16]. Mohammadi et al. [17] encapsulated *Cinnamomum zeylanicum* essential oil (CEO) by chitosan nanoparticles (CSNPs) and found that the shelf life of cucumbers with CEO-CSNPs coating was extended up to 21 days at 10 ± 1 ˚C while uncoated fruit were unmarketable in less than 15 days. Mulla et al. [18] coated chromic acid-modified linear low-density polyethylene (LLDPE) surfaces with clove essential oil (CLO) and observed their strong antimicrobial activities against *S*. *typhimurium* and *L*. *monocytogenes* in a packed chicken sample for 21 days of refrigerated storage.

Among the different EOs, lemon essential oil (LEO) and tea tree oil (TTO) exhibited significant inhibitory effects against bacteria [19, 20], especially TTO showing high antimicrobial efficacies against various antibiotic-resistant *Staphylococcus* species [19]. This study focuses on the incorporation of TTO or LEO coatings to MWCNTs filters to achieve highly effective bacterial removal from aqueous solutions and complete inactivation of captured bacteria on the filters under optimal conditions, using laboratory model bacteria *E*. *coli*

## Materials and methods

### Preparation of MWCNTs filters, TTO-MWCNTs filters, and LEO-MWCNT filters

MWCNTs were purchased from NanoIntegris Inc. (Skokie, IL, USA). According to the manufacturer, these MWCNTs had the purity of > 95 wt% and the length of 10–30 μm. The specific surface area was 233 m^2^/g with 10–20 nm and 3–5 nm on the outer and inner diameter, respectively. The preparation procedure of MWCNTs filters was similar as our previous study [13]. Briefly, the as-received MWCNTs were suspended in 50% dimethylsulfoxide (DMSO) at the concentration of 3 mg/mL. The MWCNTs suspensions were sonicated for 15 min and then deposited onto the Isopore^TM^ RTTP membranes filters with a diameter of 25 mm and a pore size of 1.2 μm (Merck Millipore Ltd., Carrigtwohill, Ireland) with various loadings by varying the depositing volumes. The membranes loaded with MWCNTs were sit at room temperature for 2 h, and were washed by filtering with 2 mL ethanol first and then 5 mL deionized water (DI-H_2_O).

TTO and LEO were purchased from Sigma-Aldrich (St. Louis, MO, USA). For the preparation of TTO-MWCNTs filters and LEO-MWCNTs filters, various volumes of TTO or LEO was added onto the surface of MWCNTs filters to coat MWCNTs. Mixture of 1:1 (v/v) TTO and LEO also used to coat MWCNTs filters, and denoted as Mix-MWCNTs filters. The filters were then washed with 5 mL DI-H_2_O to remove the un-adsorbed EO and were used immediately.

### Characterization of the EO-incorporated filters

The hydrophilic properties of MWCNTs, TTO-MWCNTs, and LEO-MWCNTs filters were characterized by measuring the water contact angles of the filters using the water sessile drop methods on a goniometer (Ramé-hart instrument co., Succasunna, NJ, USA). The mean contact angles were calculated based on a drop of DI-H_2_O on the surface with five replicates.

The surface morphologies of filters were examined using the scanning electron microscopy (SEM) at the Shared Materials and Instrumentation Facility of Duke University.

### Determination of bacterial removal and inactivation efficiencies of the resulting filters

*E*. *coli* cells were grown in Luria-Bertani (LB) broth at a shaker under 37˚C overnight. The cells were washed and diluted to the concentration of ~ 10^7^/ml in PBS. Aliquots of 2 ml cell suspensions were filtered by MWCNTs, TTO-MWCNTs, or LEO-MWCNTs filters at a flow rate of 0.2 ml/min using a syringe pump. The filtrates were collected, serial diluted with PBS, and surface plated on LB agar plates. The plates were incubated at 37˚C for 18 h, and the viable cell numbers were counted and calculated in colonies forming units per milliliter (CFU/ml). Log reduction in viable cell number after filtration was used to indicate how effective the filters were for bacterial removal. In some cases, the bacterial removal efficiency by the filters were also calculated using the following formula:

Bacterial removal rate (%) = (Initial cell numbers before filtering—Cell numbers in the filtrate) ×100% / Initial cell numbers before filtering.

To determine the inactivation efficiency to bacterial cells captured on the filters, after the filtration step, the filter membranes were unassembled, and the captured cells on the filter membranes were washed off from the surfaces by immersing the filters into 2 mL PBS and vortexing vigorously for 2 min. The viable cell numbers in the suspensions were determined by the surface plating method using the same procedure described above. The inactivation efficiency was calculated using the following formula, where the total captured cell number was calculated using the initial cell number subtract the cell numbers in filtrate.

Inactivation efficiency (%) = (Total captured cell number- Viable cell numbers of captured cells) ×100% /Total captured cell number

### Salt tolerance loss analysis on captured cells

TTO-MWCNTs filters loaded with 5.4 mg MWCNTs and 20 μL TTO were used to filter and capture the cells, and the salt tolerance of the survived bacterial cells captured on the filters’ surfaces was evaluated. In order to compare the salt tolerance of the captured cells on TTO-MWCNTs filters, bacterial cell samples filtered by uncoated membranes (control), MWCNTs (5.4 mg) filters, and TTO (20 μL) coated membranes were also evaluated for their salt tolerance. Briefly, the bacteria cells in PBS were first filtered and retained on the filter surfaces for 0–2 h at the room temperature, and then the cells were removed from the filter surfaces, serial diluted, and cultured on LB agar plates containing 0, 30, or 40g/L of NaCl. For each type of filter, the cells on LB agar plates without NaCl were used as the control. The salt tolerance of the cells were evaluated by the percentage of salt tolerant cells within the total cells, which was calculated using the cell numbers on the plates containing 30 or 40g/L of NaCl divided by the cell numbers on the control plates without NaCl.

### Statistical analysis

Statistical analyses were performed to demonstrate the effects of different filters by using the general linear model (GLM) procedure of the SAS System 9.2 (SAS Institute Inc., Cary, NC, USA). It was considered as a significant difference when *P* < 0.05.

## Results and discussion

### The effect of MWCNTs loading on the bacterial removal efficiency of the resulting MWCNTs filters

**Fig 1:**
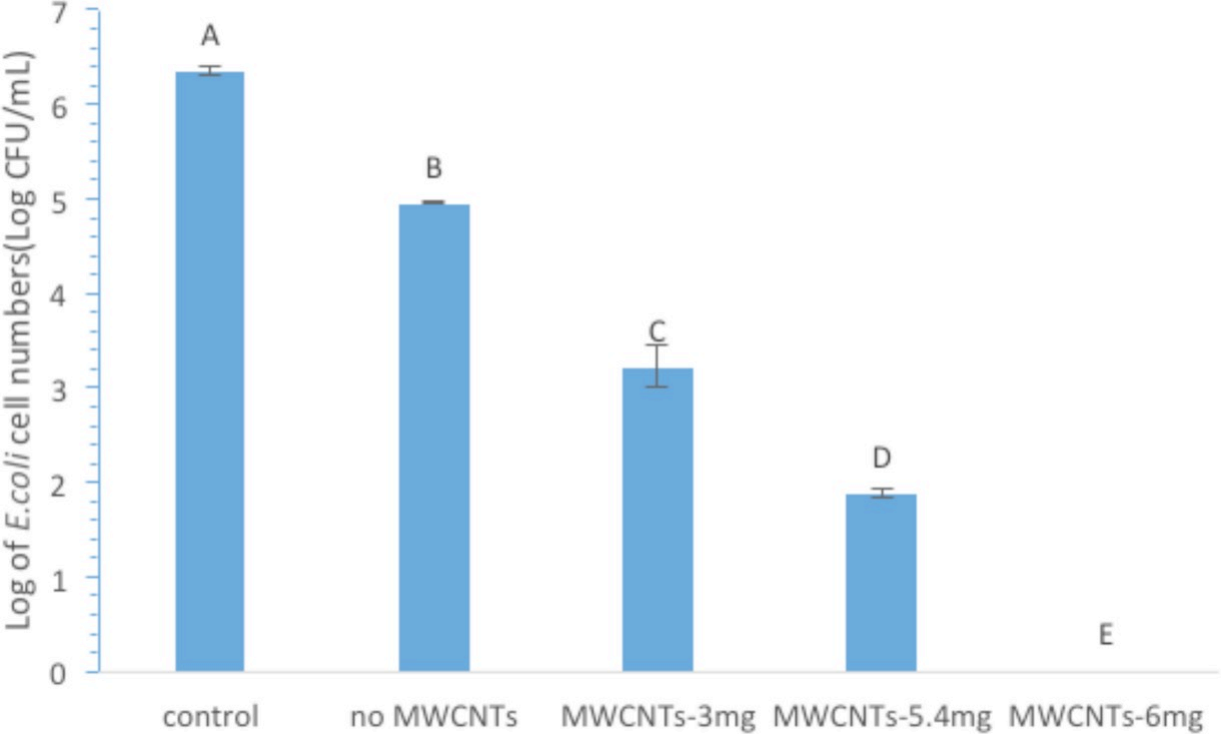
Log of *E*. *coli* cell numbers in filtrates after filtering through MWCNT filters with different MWCNT loadings. **Notes:** Statistical analysis results are indicated by the letters on the bars. Different letters above the bars indicate statistical difference between the results (P<0.05); identical letters above the bars indicate no statistical difference.

MWCNTs filters with different MWCNT loadings were prepared and evaluated for their bacterial removal efficiency. To prepare MWCNTs filters with different MWCNT loadings, 1000, 1800, or 2000 μL of 3mg/mL MWCNT suspension was loaded onto the RTTP membranes and followed the procedures described above to produce MWCNTs-filters with 3.0 mg, 5.4 mg, and 6.0 mg MWCNTs loadings. The RTTP membranes without any coating were used as the controls for comparison. The resulting MWCNTs filters were used for filtering *E*. *coli* cells in PBS suspension. Fig 1 shows the results of the logarithmic cell numbers in the filtrates after the bacterial samples were filtered by these filters with different MWCNT loadings. The cell numbers were determined by the surface plating method, and the log reduction in cell number compared to the control indicated how effective the filters were for removing bacterial cells from the solutions. As shown in the Fig 1, the filters with the MWCNT loading of 0, 3.0, 5.4, and 6.0 removed 1.40, 3.14, 4.48, or 6.35 log of *E*. *coli* cells, respectively. The loading of 6.0 mg MWCNTs completely removed bacterial cells from the suspensions, showing 100% bacterial removal, and all MWCNT filters significantly (P<0.05) removed *E*. *coli* cells from the solutions. Clearly, the bacterial removal efficiency of the filter was dependent on MWCNTs’ loading, with higher MWCNTs loading having higher efficiency of cell removal. The loading of MWCNTs was obviously proportional to the coverage and density of MWCNTs on the membrane, which attributed to the reduced pore sizes on the resulting membranes. Most importantly, the high adsorption property of MWCNTs to bacterial cells attributed to the high bacterial capture character of the MWCNT filters. In fact, MWCNTs’ high affinity to bacteria and other microorganisms have been manifested in different filter settings reported by others [21]. For example, MWCNTs/Trix buckypapers prepared on a 5 μm pore sized PTFE membranes could remove > 99% (equivalent to 2 log reduction) of *E*. *coli* cells in 0.9% NaCl solution [21]. Vecitis et al. found that anodic MWCNTs microfilters in the absence of electrolysis showed a complete removal of bacteria by sieving and multilog removal of MS2 viruses by depth-filtration [22]. It is also worth to note that the adsorption kinetic of CNTs to microorganisms was strongly dependent on the microorganism species involved, for example, the smaller size *S*. *aureus* has a five to ten times faster diffusion rate than *E*. *coli* and about 100 times higher adsorption affinity with the CNTs aggregates [23].

### The effect of EO coating on the bacterial removal efficiency of the resulting EO-MWCNTs filters

Most relevant to this study is MWCNTs’ high absorptivity to oils. MWCNTs have the function to effectively adsorb oils due to their exceptional absorptivity, large surface area, and oleophilic and hydrophobic nature [24], as such MWCNTs have been used to remove oils from contaminated water. For example, Wang et al. [25] added magnetic MWCNTs into the oil-water emulsion, in which MWCNTs were attracted to the oil-water interfaces, and physically removed MWCNTs-wrapped oil droplets from water by the application of a magnetic field. It is noted that the oil removal efficiency were dependent on the different initial oil concentration, MWCNTs dose, and mixing time. The kinetics and equilibrium of the oil removal process could be described by the Langmuir model [25]. Fard et al. [26] reported that high quality CNT bundles showed oil adsorption capacity of more than 7 g/g and have high efficiencies of oil removal in water, displaying 97% removal rates by P-CNTs and 87% removal rates by C-CNTs.

Taking advantage of MWCNTs’ strong absorptivity to oils, this study used the MWCNT filters to incorporate LEO and TEO to afford the filters with additional antimicrobial function. To examine if the EO coating on MWCNT filter surface affected bacterial removal rates, TTO at the volume of 10, 20, or 50 μL, LEO at the volume of 50 μL, and the mixture (Mix) of 25 μL TTO and 25 μL LEO were used to coat on MWCNT filter surfaces with 6 mg MWCNTs loading. Table 1 shows the bacterial removal rates of the MWCNTs filters incorporated with different amount of TTO, LEO and the mixture of TTO&LEO. The bacterial removal rates of these filters were calculated based on the cell numbers in the samples before and after filtration using the formula presented in Materials and Method. As shown in Table 1 when the TTO coating increased from 0 to 50 μL, the average bacterial removal rates of the resulting TTO-MWCNT filters decreased from 100% to 98.78%, similar magnitudes of decreases were observed in the LEO-MWCNTs filters and Mix-MWCNTs filters, indicating the EO coating on MWCNTs slightly reduced bacterial removal rates compared to the MWCNTs filters without EO coating.

**Table 1:** Effect of essential oil TTO and LEO coating of MWCNT filters on bacterial removal rates.

Filter type	Essential oil coating	Bacterial removal rate (%)
MWCNTs	0	100 ± 0
TTO-MWCNTs	10 μL TTO	99.08 ± 0.23
TTO-MWCNTs	20 μL TTO	98.73 ± 0.20
TTO-MWCNTs	50 μL TTO	98.78 ± 0.13
LEO-MWCNTs	50 μL LEO	98.24 ± 1.23
Mix-MWCNTs	25μL TTO + 25μL LEO	98.28 ± 0.24

**Fig 2:**
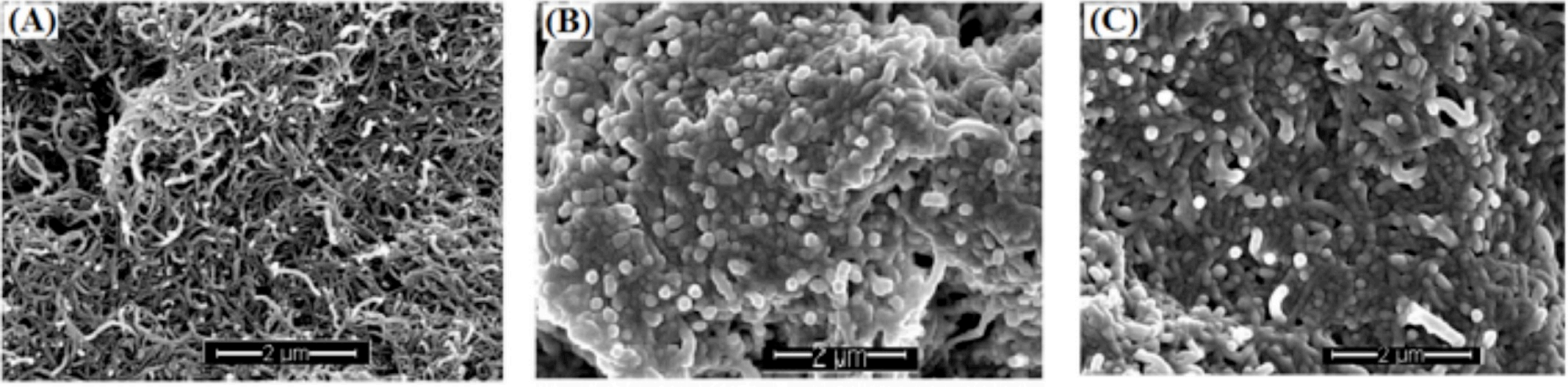
Representative SEM images of surfaces of (A) MWCNTs filter, (B) LEO-MWCNTs filter, and (C) TTO-MWCNTs filter.

The slightly decreased efficiency of EO-incorporated MWCNTs filters is likely associated with the changes in surface hydrophobicity due to EO coating, which affects bacteria attachment to it. The average contact angles of the surface of MWCNTs filters with 5.4 mg MWCNTs loading were 82.07˚, indicating slightly hydrophilic surfaces. The subsequent coating with 50 μL TTO and LEO slightly decreased the contact angles of the resulting filter surfaces by 5.52% and 7.63%, respectively, making the EO coated surface slightly more hydrophilic. The changes in surface hydrophobicity might affect bacteria attachment on the filters, leading to the slightly less effective of bacteria catpure on TTO-MWCNTs, LEO-MWCNTs, and Mix-MWCNTs filters than on the non-coated MWCNTs filters. Certainly, other surface properties, such as surface structure, surface morphology, and surface roughness, could also play roles on bacteria adsorption on the EO-MWCNTs filters. Fig 2 shows the changes in surface morphology of MWCNTs filters after coating with 50μL LEO and 50 μL TTO. As shown in the images, compared to the fluffy texture of MWCNTs filters’ surface (Fig 2A), the surfaces of LEO-MWCNTs filters (Fig 2B) and TTO-MWCNTs filters (Fig 2C) showed that the nanotubes are obviously coated with a layer of EO and displayed tighter contacts and much less spaces among the tubes. The coating of EO on MWCNTs modified the surface properties of MWCNTs and might slightly decrease the capability of MWCNTs for bacterial attachment. In literature, changes in the arrangement and properties of nanoparticles by EO coating have been reported [27]. Predoi et al. [27] observed that basil and lavender EO coatings changed the morphology of the hydroxyapatite nanocomposites (HAp) into agglomerated particles and altered the HAp’s physic-chemical properties. It’s also known that nano- and micro- scale surface roughness enhances the adhesion of bacteria to substrates as it provides more surface area for cell attachment [28]. Taking together, TTO and LEO coatings on MWCNTs obviously changed the surface properties of MWCNTs, including but not limited to surface hydrophobicity, morphology, roughness and other physic-chemical properties, which led to the slightly reduced capability of EO-MWCNTs filters for bacteria capture, while additional antibacterial function of the filters by EO coating is expected.

### The inactivation effect of EO-MWCNTs filters on the captured bacteria

**Fig 3:**
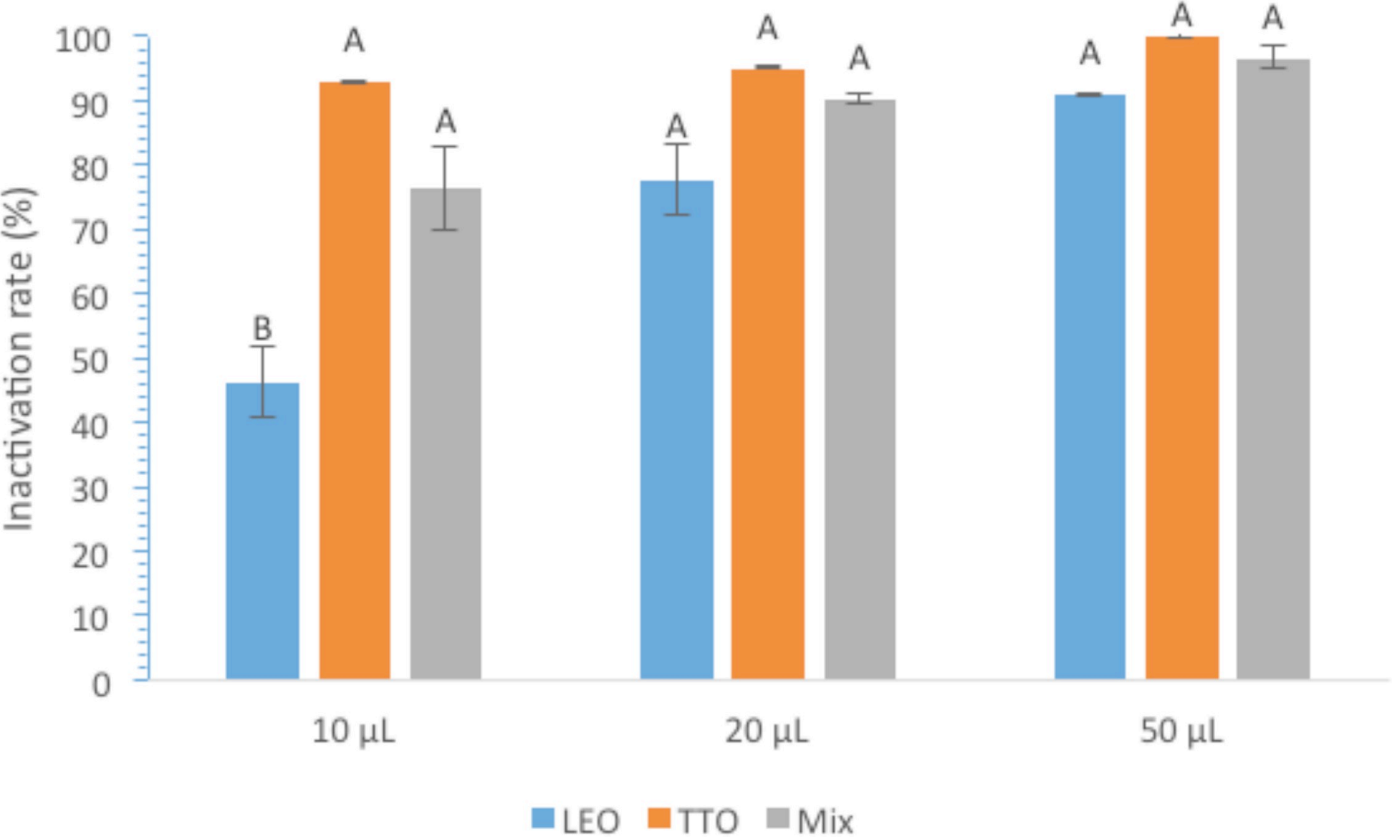
Inactivation rates of *E*. *coli* bacteria retained on the surfaces of LEO-MWCNTs, TTO-MWCNTs, or Mix-MWCNTs filters with 10, 20 or 50 μL EO coatings. **Notes:** Statistical analysis results are indicated by the letters on the bars. Different letters above the bars indicate statistical difference between the results (**P**<0.05); Identical letters above the bars indicate no statistical difference.

The effects of EO coating on the inactivation of bacteria captured on the filter surfaces were studied using the filters coated with LEO, TTO, or Mix (half LEO and half TTO) at the volume of 10, 20, or 50 μL on the base MWCNTs filter loaded with 5.4 mg MWCNTs. Fig 3 shows the results of inactivation efficiencies of the cells that were captured on the filter surfaces after the filtering step and were allowed to sit at the room temperature for 15 min before the culture procedures. As shown in Fig 3, among the LEO-MWCNTs, TTO-MWCNTs, and Mix-MWCNTs filters, with 20 μL EO coating, the inactivation rates were 77.5%, 95.0%, and 90.2%, respectively, similar trends were observed on the filters with 10 μL and 20 μL EO coating, indicating that TTO coatings exhibited the highest bacterial inactivation rate compared to LEO and the Mix coatings, despite the inactivation efficiencies of most of these filters were not statistically different except the filters with 10 μL LEO coating. The higher antibacterial effects of TTO than LEO were also observed by other studies, in which EOs were added with synthetic preservatives in cosmetics (oil in water body milks) [29].

Within the same type of EO coatings, the inactivation rate increased with the increasing amount of EO coating, as the average inactivation rates on TTO-MWCNTs filters with 10, 20, and 50 μL TTO coating were 92.80%, 94.96%, and 100%, respectively. Noticeably, TTO coating at the volume of 50 μL completely inactivated all bacterial cells. The highly effective inactivation function of EO-MWCNTs filters is expected since the antimicrobial properties of EOs have been observed in many studies [15, 30, 31]. For example, Li et al. [32] reported the antimicrobial activities of TTO to bacteria and fungal, and the minimum inhibitory concentrations of TTO for bacteria (*E*. *coli* and *S*. *aureus*) and fungal (*C*. *albicans* and *A*. *niger*) were 1.08 and 2.17 mg/mL, respectively.

The antimicrobial efficacy of a given EO depends on its chemical composition, such as its phenolic components [30, 31]. TTO is reported to contain more than 100 components, in which the main ingredients are terpene hydrocarbons, such as monoterpenene, sesquiterpenens, and their associated alcohols. The most abundant component is terpinen-4-ol, accounting for approximately 41.3% of the TTO [29]. TTO and its components, terpinen-4-ol, terpinolene, α-terpinene, and α-terpineol, had strong inhibitory activities against *S*. *aureus* [33]. Terpinene-4-ol was also observed to be the most active ingredient of TTO to kill Demodex mites, showing more potent than TTO at equivalent concentrations, displaying killing effects even at a mere concentration of 1% [34]. When terpinene-4-ol plasma polymer films were fabricated by PECVD, they could inhibit the early stages of biofouling and showed antifouling activity in seawater for one week, indicating that the possible mechanism of the effects was due to the dissolution of the film coupled with antimicrobial properties of terpinen-4-ol [35]. As for LEO, it contains different chemical compositions–the reported commercial LEO purchased from the manufacturer contained limonene 79.8%, β-pinene 3.6%, γ-terpinene 2.3%, geranial 2.7%, and neral 1.0% [29]. The lower antibacterial activity of LEO might be due to the different chemical compositions and relatively lower antimicrobial component content when compared with TTO. It is known that the concentrations of the components of EOs, even of the same type of EOs from the same plant species, can vary significantly due to the different plant location, different plant sections of extraction, and different preparation conditions, and these differences may influence the antimicrobial activities of EOs [36].

Since the coating of EO on MWCNTs filters is also dependent on the loading of MWCNTs, the loading of MWCNTs would be indirectly affect the bacterial inactivation efficiency of the resulting filters. With this regard, the inactivation efficiencies of the filters with 7.5 mg MWCNTs loading with the coating of 50 μL LEO, TTO, or Mix were tested to be 91.55± 10.38%, 99.41 ± 0.17%, and 99.76 ± 0.07% for their bacterial inactivation efficiencies. Compared to the inactivation efficiencies of the filters with the same EO coating on the lower MWCNTs loading (5.4 mg) (in Fig 3, the results indicated the filters with higher MWCNTs loading (7.5 mg) had slightly higher inactivation rates on the captured *E*. *coli* bacteria than those of lower MWCNTs loading (5.4 mg). However, with TTO coatings, filters with 5.4 mg and 7.5 mg MWCNTs loadings both exhibited 100% inactivation rates. It is understood that the filters with higher MWCNTs loadings had a larger total surface area and higher capacity to adsorb more EO, which explains the observed higher inactivation rates on filters with higher MWCNTs loadings, except the TTO coated ones which also had sufficient TTO on the filters of 5.4 mg MWCNTs loading. Other studies also observed that CNTs at higher dosages adsorbed more oils from water due to the larger number of available spaces of CNTs [26], though many other factors such as CNT’s surface area, zeta potential, aspect ratio, contact angle, dosage, contact time, etc. have various impact on the adsorption of oil on the surface of CNTs [26]. Among the factors, large surface area can be one of the major factors to increase the adsorption capacities of CNT-based surface structures, for which Jin et al. [37] demonstrated excellent adsorption capacities of three-dimensional flower-like CNTs-nanofibers foams to selectively adsorb oils and organic liquids. Therefore, the optimal combination of MWCNTs loading and EO coating could achieve the goal to completely remove bacterial cells from aqueous solutions and completely inactivate the cells retained on the filters.

### The effect of incubation time on inactivation rate of bacteria on LEO-MWCNTs filters

By using the filters loaded with 3 mg MWCNTs and 50 μL LEO, the inactivation rates at different incubation time on the bacterial cells retained on top of the filter surfaces after the filtering step were tested. The results indicated that the antimicrobial effects of LEO coating were effective immediately after the filtering step and slightly increased with the increasing incubation time from 0 to 3 h. As shown in Table 2, although the LEO-MWCNTs filters did not completely inhibit bacterial growth even after 3 h incubation, the inactivation rates were clearly increased with the incubation time, with the average inactivation rate increased from 90.01%, to 97.03%, and 98.47% at the incubation time of 0, 30 min, and 60 min, respectively, and increased to >99% at incubation times of 2 h and 3 h.

**Table 2:** Inactivation rate of the LEO-MWCNTs filters to *E*. *coli* cells captured on the filters with different incubation time after the filtering step.

Contact time	Inactivation rate (%)
0	90.01 ± 0.17
0.5 h	97.03 ± 2.6
1 h	98.47 ± 0.09
2 h	99.49 ± 0.12
3 h	99.54 ± 0.21

In summary, the EO-MWCNTs filters showed much stronger and quicker antimicrobial effects than many antimicrobial coating surfaces developed by our previous studies and by others. For example, our previously developed MWCNTs filters with 0.2 mg carbon dots (CDots) or 0.2 mg SWCNTs coating needed 30 min contact time to inactivate 94.2% or 73.2% of *E*. *coli* cells, respectively [4]. Humblot et al. used an antibacterial peptide, Magainin I, to covalently bind onto mixed thiols self-assembled monolayer on gold surfaces [38]. Their results showed that 30 min contact time with grafted Magainin I did not alter the adhered bacterial numbers, and the time of 3 h contact only reduced 50% of the adhered cell numbers [38]. Walker and coworkers developed a light-activated antimicrobial surface–a silicone incorporating crystal violet and methylene blue and 2 nm gold nanoparticle, which showed 2.92 log10 reduction after 3 h treatment, but did not show significant reduction in *S*. *epidermidis* population after 1 h and 1.5 h treatment [39]. Thian et al. developed zinc-substituted hydroxyapatite (ZnHA) as an antibacterial biomaterial and observed that the bacterial numbers showed one log10 bacterial cell number increase on ZnHA after one day contact, and 2 log10 reduction after 3 days incubation [40].

### Bacterial morphological changes on LEO- and TTO-MWCNTs surfaces and the possible antimicrobial mechanisms

**Fig 4:**
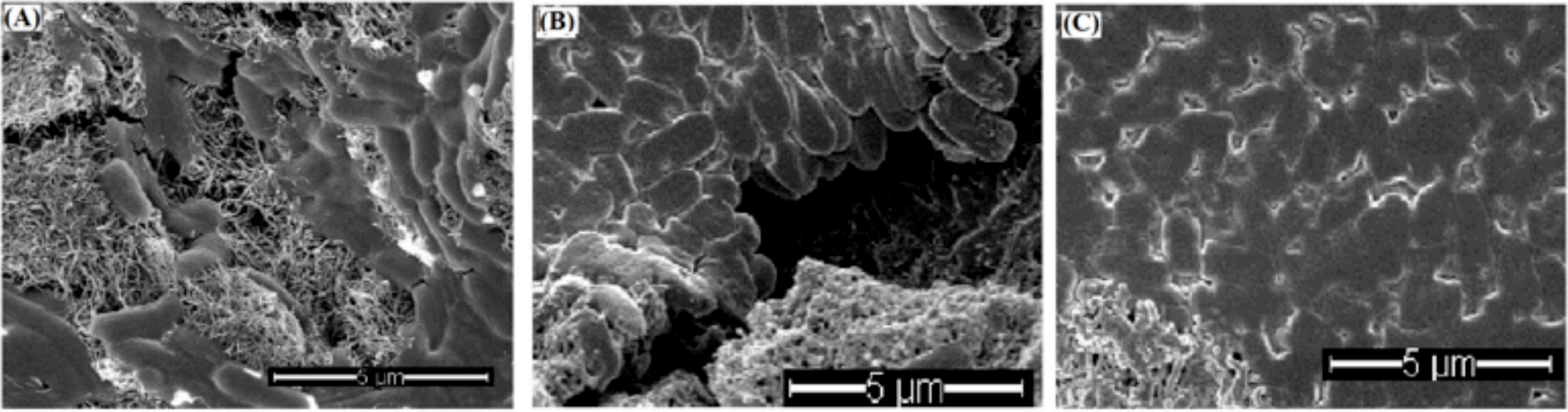
SEM images of *E*. *coli* cells on (A) a MWCNTs filter, (B) a LEO-MWCNTs filter, and (C) a TTO-MWCNTs filter. The filters were prepared by coating 50 μL LEO and 50 μL TTO on MWCNTs filters.

The antimicrobial mechanisms of EO have been largely studied and discovered that the mechanisms of action between different EOs had similarities and differences with each other. Studies reported that TTO penetrated the cell wall and cytoplasmic membrane of all the TTO treated bacterial and fungal strains according to the transmission electron microscopy (TEM) images, indicating that TTO maybe exert its antimicrobial effects by compromising the cell membrane and causing the loss of cytoplasm and cell deaths [32]. Other studies found that the penetration of EOs into the cytoplasmic membrane was due to the lipophilicity [41, 42]. When *S*. *aureus* bacteria were treated with terpinen-4-ol at MIC and two times MIC, the cells became sensitive to subsequent autolysis, and their structure showed morphology changes, the formation of mesosome, and the loss of cytoplasmic contents [43]. EOs of *Ageratum conyzoides* could cause ultra-structural changes, especially in the endomembrane system, in pathogenic fungus *Aspergillus flavus*, affecting mainly the mitochondria [41]. Xiang et al. [44] found that EO from the traditional Chinese herb Qiai caused disruption of cell structures, such as the cytomembrane, leading to the leakage of intracellular substances such as protein and K^+^, and causing cell death. With this regard, morphological changes in *E*. *coli* cells retained on LEO-MWCNTs and TTO-MWCNTs filters were examined by SEM imaging (Fig 4). Most bacteria on the surface of MWCNTs filter were intact and full in shape (Fig 4A), while the bacteria on the surfaces of LEO-MWCNTs filter (Fig 4B) and TTO-MWCNTs filter (Fig 4C) were flat, especially the cells on the TTO-MWCNTs filter. A large number of bacteria on the TTO-MWCNTs filter were flat with irregular or unclear, merged outlines, showing severe cell structure damages. The change of cell morphology indicated that both LEO-MWCNTs and TTO-MWCNTs filters damaged the cell walls and cell membranes, caused cytoplasmic contents leakage, and led to the cell appearance of flat shape and irregular outlines.

Bacterial morphological changes were a common phenomena observed on other types of antimicrobial surfaces. For instance, Walker et al. treated bacterial cells on a light-activated antimicrobial silicone surfaces for 2.5 h with light and observed irregularly shaped indentations on *S*.*epidermidis* bacterial surface, suggesting an interaction between the cells and the surfaces [39]. Bacteria on copper surfaces showed cellular structure damages by released copper ions, leading to a loss of membrane potential and cytoplasmic content [45]. Fisher et al. reported fabrication of nanocone-shaped diamond on silicon substrate and observed cell membrane damages and bacteria lysis [46]. Other nanowire-modified antimicrobial filters based Ag+-induced bacterial inactivation or electroporation-inactivation also resulted in bacterial morphological changes [47, 48]. Our LEO- and TTO- MWCNTs filters showed much more severe damages on captured bacteria than the antimicrobial surfaces from these literatures, especially our TTO(50 μL)-MWCNTs filters, which damaged all the captured bacteria and showed 100% inhibitory rates.

### Loss of NaCl tolerance of bacteria captured on EO-MWCNTs filters

**Fig 5:**
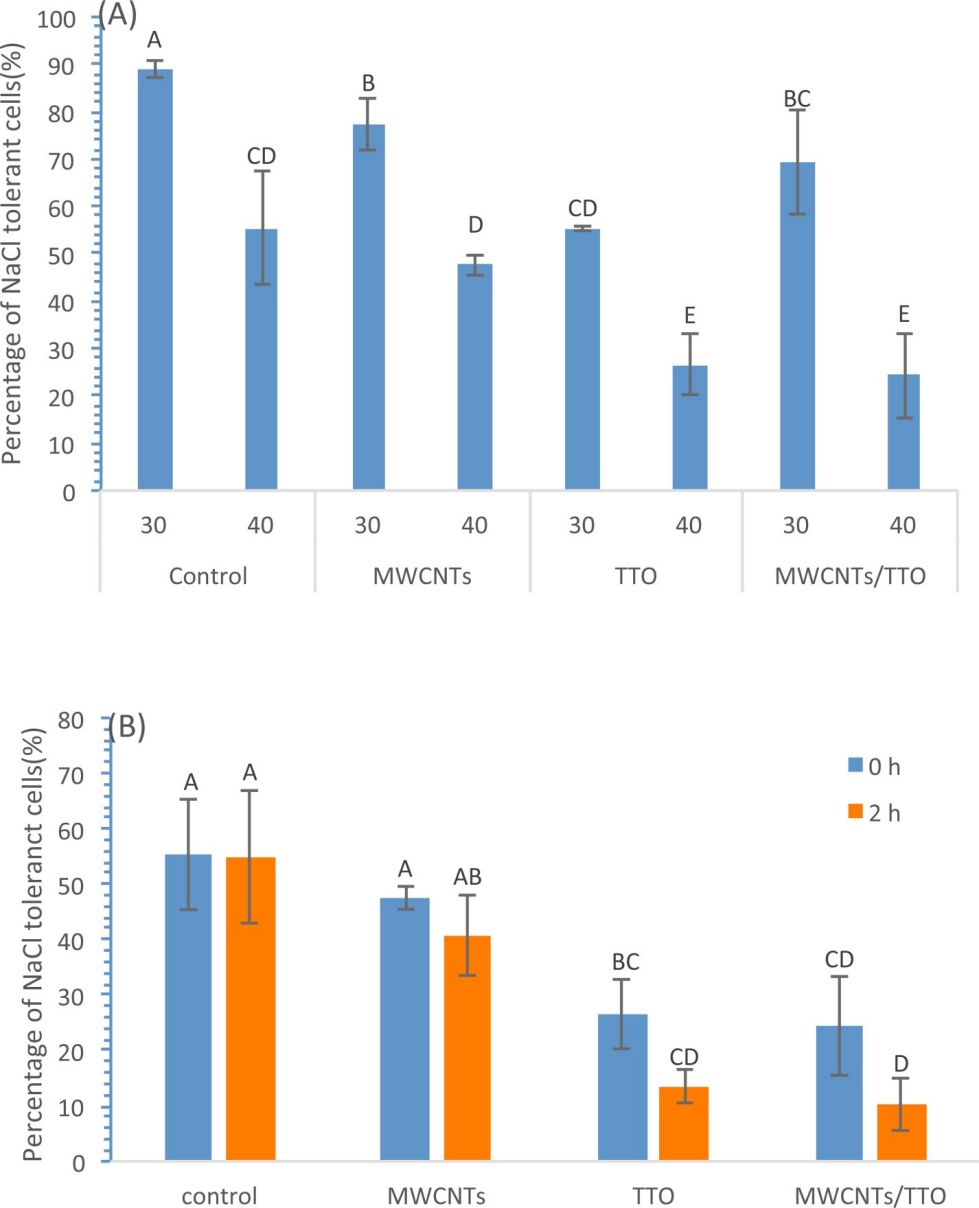
The percentage of NaCl tolerant *E*. *coli* cells retained on filters. (A) The cells without incubation step on the filters were immediately tested on LB agar plates supplemented with 30 and 40 g/L NaCl. (B) The cells after 0 and 2 h incubation on the filters were tested on LB agar plates supplemented with 40 g/L NaCl. **Notes:** Statistical analysis results are indicated by the letters on the bars. Different letters above the bars indicate statistical difference between the results (P<0.05); Identical letters above the bars indicate no statistical difference.

Bacterial morphological changes are usually associated with membrane damages and subsequent leaking of cytoplasmic contents and loss of various bacterial activities. It was reported that many EOs such as TTOs compromise bacterial cytoplasmic membranes, leading to the loss of NaCl tolerance on bacteria [43]. To test if EO coatings on MWCNTs filters changed the cells’ salt tolerance and to better understand the filters’ antimicrobial mechanisms, the survived bacterial cells retained on filters’ surfaces were used to test their NaCl tolerance. Fig 5(A) shows the percentage of NaCl (30 and 40 g/L) tolerant cells captured on filters’ surfaces immediately after the filtering step. The percentage of the NaCl tolerant cells in the total survived cells on TTO-MWCNTs filter surface was 69.23% and 24.36% under 30 g/L and 40 g/L NaCl, respectively, and the cells showed a significant lower percentage in NaCl tolerance under 40 g/L NaCl than under 30 g/L (P < 0.05) on each type of filters. The percentage of NaCl tolerant cells on MWCNTs, TTO, or TTO-MWCNTs filters were significantly lower than the counterparts of the control samples (P < 0.05). The percentage of NaCl tolerance cells on TTO coated filter membranes (TTO only, or TTO-MWCNTs) were similar (P > 0.05) as those of the counterparts on TTO-MWCNTs filters and were significantly lower (P < 0.05) than those of counterparts on the control filters, indicating TTO coating immediately decreased the bacterial NaCl tolerance. This observation was correlated with the results above that TTO coating immediately inactivating the bacteria’s activities.

To test if the incubation time on the filter surfaces could affect the NaCl tolerance of bacteria, an assay was performed by incubating the cells on the filter surface for 0 or 2 h at room temperature and then culturing the cells on LB agar plates supplemented with 40 g/L NaCl. Fig 5(B) shows that the percentage of salt tolerant cells decreased from 26.56% to 13.57% on TTO filters and from 24.36% to 10.23% on TTO-MWCNT filters with 2 h incubation, indicating that the 2 h incubation step decreased the NaCl tolerance of *E*. *coli* cells retained on EO-MWCNTs filters compared to the control samples. Taking together with the results observed above that longer incubation time on LEO-MWCNTs filters caused higher inactivation rates of retained bacteria, these observations suggested that the longer contact time of bacteria with EO coated surfaces may cause changes in bacterial membrane permeability which resulted in decreased bacterial viability and salt tolerance.

Overall, our observations showed similar trends to the results reported by other researchers, except that the captured bacteria in this study responded more quickly to and showed less tolerance on high NaCl concentrations. For instance, *E*. *coli* cells need a 30 min-treatment of 0.22% *Kaempferia pandurata* EO in DI-H_2_O to significantly reduce their ability to form colonies on nutrient agar (NA) plates supplemented with 50 or 75 g/L NaCl, displaying 13% or less of the survivors able to form colonies [49]. *S*.*aureus* cells needed 30 min treatments of TTO or its components at two times their MICs in PBS supplemented with 0.001% Tween 80 (PBS-T) to significantly reduce their ability of forming colonies on NA plates with 50 g/L or 75 g/L NaCl, showing 1.5% or less of the survived cells able to form colonies [43]. In this test, the captured cells on TTO and TTO-MWCNTs filters immediately and completely (100%) inhibited the cell growth on LB agar plates with 50 g/L NaCl. Compared to bacteria treatments with EO in solutions, the bacteria cells captured on the filter surfaces had contacts with much higher TTO concentrations at the cell-surface contact sites, causing more severe local damages on cell structures and leading to deaths or quicker response and loss tolerance to high NaCl conditions.

## Conclusions

This study demonstrated that EO coated MWCNTs filters, especially TTO coated MWCNTs filters, were highly effective on bacteria removal and inactivation. At the optimal combination of MWCNTs loading (6 mg/mL) MWCNTs and TTO coating (50 μL), the filters removed 100% *E*. *coli* cells from PBS suspensions, and inactivated all the cells captured on the filters, showing >6 log reduction of cell numbers. LEO and TTO-LEO-Mix coatings on MWCNTs filters also achieved very high inactivation efficiencies at 99%. Both TTO and LEO coatings on the MWCNTs filters caused the significant loss of NaCl tolerance of captured survival bacteria on the filters. The mechanisms of action of EO coated MWCNTs filters to inactivate bacterial were mostly through the severe damages of bacterial cell walls and cell membranes, leading to the loss of cytoplasmic content and cell deaths. The results of this study demonstrated the dual functions of EO coated MWCNTs filters in highly effective bacteria removal and inactivation, which may be advanced for potential applications to remove and/or inactivate bacterial pathogens in water to improve water quality and safety.
