# Supplementary material for: Essential oil-incorporated carbon nanotubes filters for bacterial removal and inactivation
Source: PLoS One. 2019 Dec 27;14(12):e0227220. doi: 10.1371/journal.pone.0227220 (PMC6934282; doi:10.1371/journal.pone.0227220)
Supplement: Attachment — Submitted filename: Responses to Reviewers Comments-Dec2019.docx [file pone.0227220.s001.docx]

**Manuscript #:** PONE-D-19-29053

**Title:** Essential Oil-Incorporated Carbon Nanotubes Filters for Bacterial Removal and Inactivation

**Responses to Editor comments:**

1. A rebuttal letter addressing all the reviewer comments is included.
2. A mark-up copy of the revised manuscript is included.
3. An unmarked version of the revised manuscript is included.
4. ORCID iD is linked.
5. Table 2 is included. (It was incorrectly numbered as Table 3 and it is now corrected).

**Responses to Review Comments 1:**

Reviewer #1: The authors presented a new antimicrobial material, essential oil-incorporated CNT and studied its disinfection efficiency against bacteria. The manuscript provided enough scientific results and abundant discussion. It can be considered to accept for publication after the authors address the following questions.

1. The authors used two quantitative methods for bacterial counting: cell removal rate and inactivation efficiency. The description is clear in the Materials and Methods part. However, in the Results and Discussion, multiple different names are used, especially in the figures, such as "log10 of E.coli cell number", "bacterial removal rate", "inhibition rate", and "rate of NaCl tolerance cells". Please clarify or clearly mark how these numbers are measured (by cell removal rate or inactivation efficiency).

**Our Response:** We greatly appreciate the reviewer’s comment on this. We have now revised the terms in the text, and have them explained or matched in Materials & Methods and in Results & Discussion.

2. In figure 1, the authors uses "log" to present the inactivation by CNT independently. In the following figures, the authors uses "%", which indicates a lower removal/inactivation efficiency. So is this meaning that the incorporation of essential oil decreases the inactivation efficiency and hinder the antimicrobial power of CNT?

**Our response:** There are two functions of filters: removal of bacterial cells from solutions, and inactivation of cells that were retained on the filters during filtration. Figure 1 used the Log reduction of cells to present ***the bacterial removal efficiency*** by CNT-filters. The % in Table 1 does mean that the incorporation of essential oil slightly decreases the bacterial removal efficiency, and an explanation for the slightly reduced removal efficiency was given in the paragraph followed. The following figure (Fig 3) using % to indicate ***the inactivation efficiency*** of the filters incorporated with essential oil. The incorporation of EO was to provide the filters with antimicrobial function.

3. The treatment capacity (flow rate) is pretty low in this study. Can the authors indicate the potential applications of this material?

**Our response:** Since it is a fundament proof of concept study, experiments were performed using laboratory syringe filters, which can only use low flow rates. But we mentioned very briefly the potential applications of this concept/materials in the Conclusion part, which is mainly for pathogen removal and inactivation to improve water quality and safety.

4. When discussing the bacterial morphological change under different antimicrobial materials, the authors could consider to include the following references:
J. Mater. Chem. A, 2018,6, 18813-18820
https://doi.org/10.1016/j.jcis.2009.07.052
Environ. Sci. Technol. 2019, 53, 13, 7504-7512

**Our response:** We have added these two references, which are ref 47 and 48.
