# Supplementary material for: Essential oil-incorporated carbon nanotubes filters for bacterial removal and inactivation
Source: PLoS One. 2019 Dec 27;14(12):e0227220. doi: 10.1371/journal.pone.0227220 (PMC6934282; doi:10.1371/journal.pone.0227220)
Supplement: Peer review history

# Peer review: Decision Letter 0

**DOI:** 10.1371/journal.pone.0227220.r001

**Authors:** Amitava Mukherjee

21 Nov 2019

PONE-D-19-29053

Essential Oil-Incorporated Carbon Nanotubes Filters for Bacterial Removal and Inactivation

PLOS ONE

Dear Dr. Yang,

Thank you for submitting your manuscript to PLOS ONE. After careful consideration, we feel that it has merit but does not fully meet PLOS ONE’s publication criteria as it currently stands. Therefore, we invite you to submit a revised version of the manuscript that addresses the points raised during the review process.

We would appreciate receiving your revised manuscript by Jan 05 2020 11:59PM. When you are ready to submit your revision, log on to https://www.editorialmanager.com/pone/ and select the 'Submissions Needing Revision' folder to locate your manuscript file.

If you would like to make changes to your financial disclosure, please include your updated statement in your cover letter.

To enhance the reproducibility of your results, we recommend that if applicable you deposit your laboratory protocols in protocols.io, where a protocol can be assigned its own identifier (DOI) such that it can be cited independently in the future. For instructions see: http://journals.plos.org/plosone/s/submission-guidelines#loc-laboratory-protocols

Please include the following items when submitting your revised manuscript:

- A rebuttal letter that responds to each point raised by the academic editor and reviewer(s). This letter should be uploaded as separate file and labeled 'Response to Reviewers'.
- A marked-up copy of your manuscript that highlights changes made to the original version. This file should be uploaded as separate file and labeled 'Revised Manuscript with Track Changes'.
- An unmarked version of your revised paper without tracked changes. This file should be uploaded as separate file and labeled 'Manuscript'.

Please note while forming your response, if your article is accepted, you may have the opportunity to make the peer review history publicly available. The record will include editor decision letters (with reviews) and your responses to reviewer comments. If eligible, we will contact you to opt in or out.

We look forward to receiving your revised manuscript.

Kind regards,

Amitava Mukherjee, ME, Ph.D.

Academic Editor

PLOS ONE

Journal Requirements:

1. When submitting your revision, we need you to address these additional requirements.

Please ensure that your manuscript meets PLOS ONE's style requirements, including those for file naming. The PLOS ONE style templates can be found at

http://www.journals.plos.org/plosone/s/file?id=wjVg/PLOSOne_formatting_sample_main_body.pdf and http://www.journals.plos.org/plosone/s/file?id=ba62/PLOSOne_formatting_sample_title_authors_affiliations.pdf

2. PLOS requires an ORCID iD for the corresponding author in Editorial Manager on papers submitted after December 6th, 2016. Please ensure that you have an ORCID iD and that it is validated in Editorial Manager. To do this, go to ‘Update my Information’ (in the upper left-hand corner of the main menu), and click on the Fetch/Validate link next to the ORCID field. This will take you to the ORCID site and allow you to create a new iD or authenticate a pre-existing iD in Editorial Manager. Please see the following video for instructions on linking an ORCID iD to your Editorial Manager account: https://www.youtube.com/watch?v=_xcclfuvtxQ

3. Please include a copy of Table 2 which you refer to in your text on page 17.

[Note: HTML markup is below. Please do not edit.]

Reviewers' comments:

Reviewer's Responses to Questions

**Comments to the Author**

1. Is the manuscript technically sound, and do the data support the conclusions?

The manuscript must describe a technically sound piece of scientific research with data that supports the conclusions. Experiments must have been conducted rigorously, with appropriate controls, replication, and sample sizes. The conclusions must be drawn appropriately based on the data presented. 

Reviewer #1: Yes

**********

2. Has the statistical analysis been performed appropriately and rigorously? 

Reviewer #1: Yes

**********

3. Have the authors made all data underlying the findings in their manuscript fully available?

The PLOS Data policy requires authors to make all data underlying the findings described in their manuscript fully available without restriction, with rare exception (please refer to the Data Availability Statement in the manuscript PDF file). The data should be provided as part of the manuscript or its supporting information, or deposited to a public repository. For example, in addition to summary statistics, the data points behind means, medians and variance measures should be available. If there are restrictions on publicly sharing data—e.g. participant privacy or use of data from a third party—those must be specified.

Reviewer #1: Yes

**********

4. Is the manuscript presented in an intelligible fashion and written in standard English?

PLOS ONE does not copyedit accepted manuscripts, so the language in submitted articles must be clear, correct, and unambiguous. Any typographical or grammatical errors should be corrected at revision, so please note any specific errors here.

Reviewer #1: Yes

**********

5. Review Comments to the Author

Please use the space provided to explain your answers to the questions above. You may also include additional comments for the author, including concerns about dual publication, research ethics, or publication ethics. (Please upload your review as an attachment if it exceeds 20,000 characters)

Reviewer #1: The authors presented a new antimicrobial material, essential oil-incorporated CNT and studied its disinfection efficiency against bacteria. The manuscript provided enough scientific results and abundant discussion. It can be considered to accept for publication after the authors address the following questions.

1. The authors used two quantitative methods for bacterial counting: cell removal rate and inactivation efficiency. The description is clear in the Materials and Methods part. However, in the Results and Discussion, multiple different names are used, especially in the figures, such as "log10 of E.coli cell number", "bacterial removal rate", "inhibition rate", and "rate of NaCl tolerance cells". Please clarify or clearly mark how these numbers are measured (by cell removal rate or inactivation efficiency).

2. In figure 1, the authors uses "log" to present the inactivation by CNT independently. In the following figures, the authors uses "%", which indicates a lower removal/inactivation efficiency. So is this meaning that the incorporation of essential oil decreases the inactivation efficiency and hinder the antimicrobial power of CNT?

3. The treatment capacity (flow rate) is pretty low in this study. Can the authors indicate the potential applications of this material?

4. When discussing the bacterial morphological change under different antimicrobial materials, the authors could consider to include the following references:

J. Mater. Chem. A, 2018,6, 18813-18820

https://doi.org/10.1016/j.jcis.2009.07.052

Environ. Sci. Technol. 2019, 53, 13, 7504-7512

**********

6. PLOS authors have the option to publish the peer review history of their article (what does this mean?). If published, this will include your full peer review and any attached files.

If you choose “no”, your identity will remain anonymous but your review may still be made public.

**Do you want your identity to be public for this peer review?** For information about this choice, including consent withdrawal, please see our Privacy Policy.

Reviewer #1: No

[NOTE: If reviewer comments were submitted as an attachment file, they will be attached to this email and accessible via the submission site. Please log into your account, locate the manuscript record, and check for the action link "View Attachments". If this link does not appear, there are no attachment files to be viewed.]

While revising your submission, please upload your figure files to the Preflight Analysis and Conversion Engine (PACE) digital diagnostic tool, https://pacev2.apexcovantage.com/. PACE helps ensure that figures meet PLOS requirements. To use PACE, you must first register as a user. Registration is free. Then, login and navigate to the UPLOAD tab, where you will find detailed instructions on how to use the tool. If you encounter any issues or have any questions when using PACE, please email us at figures@plos.org. Please note that Supporting Information files do not need this step.

# Author comment: Author response to Decision Letter 0

**DOI:** 10.1371/journal.pone.0227220.r002

11 Dec 2019

Responses to Editor comments:

1. A rebuttal letter addressing all the reviewer comments is included.

2. A mark-up copy of the revised manuscript is included.

3. An unmarked version of the revised manuscript is included. 

4. ORCID iD is linked.

5. Table 2 is included. (It was incorrectly numbered as Table 3 and it is now corrected).

Responses to Review Comments 1:

Reviewer #1: The authors presented a new antimicrobial material, essential oil-incorporated CNT and studied its disinfection efficiency against bacteria. The manuscript provided enough scientific results and abundant discussion. It can be considered to accept for publication after the authors address the following questions.

1. The authors used two quantitative methods for bacterial counting: cell removal rate and inactivation efficiency. The description is clear in the Materials and Methods part. However, in the Results and Discussion, multiple different names are used, especially in the figures, such as "log10 of E.coli cell number", "bacterial removal rate", "inhibition rate", and "rate of NaCl tolerance cells". Please clarify or clearly mark how these numbers are measured (by cell removal rate or inactivation efficiency).

Our Response: We greatly appreciate the reviewer’s comment on this. We have now revised the terms in the text, and have them explained or matched in Materials & Methods and in Results & Discussion.

2. In figure 1, the authors uses "log" to present the inactivation by CNT independently. In the following figures, the authors uses "%", which indicates a lower removal/inactivation efficiency. So is this meaning that the incorporation of essential oil decreases the inactivation efficiency and hinder the antimicrobial power of CNT?

Our response: There are two functions of filters: removal of bacterial cells from solutions, and inactivation of cells that were retained on the filters during filtration. Figure 1 used the Log reduction of cells to present the bacterial removal efficiency by CNT-filters. The % in Table 1 does mean that the incorporation of essential oil slightly decreases the bacterial removal efficiency, and an explanation for the slightly reduced removal efficiency was given in the paragraph followed. The following figure (Fig 3) using % to indicate the inactivation efficiency of the filters incorporated with essential oil. The incorporation of EO was to provide the filters with antimicrobial function. 

3. The treatment capacity (flow rate) is pretty low in this study. Can the authors indicate the potential applications of this material?

Our response: Since it is a fundament proof of concept study, experiments were performed using laboratory syringe filters, which can only use low flow rates. But we mentioned very briefly the potential applications of this concept/materials in the Conclusion part, which is mainly for pathogen removal and inactivation to improve water quality and safety.

4. When discussing the bacterial morphological change under different antimicrobial materials, the authors could consider to include the following references:

J. Mater. Chem. A, 2018,6, 18813-18820

https://doi.org/10.1016/j.jcis.2009.07.052

Environ. Sci. Technol. 2019, 53, 13, 7504-7512

Our response: We have added these two references, which are ref 47 and 48.

Attachment Submitted filename: Responses to Reviewers Comments-Dec2019.docx Click here for additional data file.

# Peer review: Decision Letter 1

**DOI:** 10.1371/journal.pone.0227220.r003

**Authors:** Amitava Mukherjee

16 Dec 2019

Essential Oil-Incorporated Carbon Nanotubes Filters for Bacterial Removal and Inactivation

PONE-D-19-29053R1

Dear Dr. Yang,

We are pleased to inform you that your manuscript has been judged scientifically suitable for publication and will be formally accepted for publication once it complies with all outstanding technical requirements.

Within one week, you will receive an e-mail containing information on the amendments required prior to publication. When all required modifications have been addressed, you will receive a formal acceptance letter and your manuscript will proceed to our production department and be scheduled for publication.

Shortly after the formal acceptance letter is sent, an invoice for payment will follow. To ensure an efficient production and billing process, please log into Editorial Manager at https://www.editorialmanager.com/pone/, click the "Update My Information" link at the top of the page, and update your user information. If you have any billing related questions, please contact our Author Billing department directly at authorbilling@plos.org.

If your institution or institutions have a press office, please notify them about your upcoming paper to enable them to help maximize its impact. If they will be preparing press materials for this manuscript, you must inform our press team as soon as possible and no later than 48 hours after receiving the formal acceptance. Your manuscript will remain under strict press embargo until 2 pm Eastern Time on the date of publication. For more information, please contact onepress@plos.org.

With kind regards,

Amitava Mukherjee, ME, Ph.D.

Academic Editor

PLOS ONE

Additional Editor Comments (optional):

Reviewers' comments:

Reviewer's Responses to Questions

**Comments to the Author**

1. If the authors have adequately addressed your comments raised in a previous round of review and you feel that this manuscript is now acceptable for publication, you may indicate that here to bypass the “Comments to the Author” section, enter your conflict of interest statement in the “Confidential to Editor” section, and submit your "Accept" recommendation.

Reviewer #1: All comments have been addressed

**********

2. Is the manuscript technically sound, and do the data support the conclusions?

The manuscript must describe a technically sound piece of scientific research with data that supports the conclusions. Experiments must have been conducted rigorously, with appropriate controls, replication, and sample sizes. The conclusions must be drawn appropriately based on the data presented. 

Reviewer #1: Yes

**********

3. Has the statistical analysis been performed appropriately and rigorously? 

Reviewer #1: Yes

**********

4. Have the authors made all data underlying the findings in their manuscript fully available?

The PLOS Data policy requires authors to make all data underlying the findings described in their manuscript fully available without restriction, with rare exception (please refer to the Data Availability Statement in the manuscript PDF file). The data should be provided as part of the manuscript or its supporting information, or deposited to a public repository. For example, in addition to summary statistics, the data points behind means, medians and variance measures should be available. If there are restrictions on publicly sharing data—e.g. participant privacy or use of data from a third party—those must be specified.

Reviewer #1: Yes

**********

5. Is the manuscript presented in an intelligible fashion and written in standard English?

PLOS ONE does not copyedit accepted manuscripts, so the language in submitted articles must be clear, correct, and unambiguous. Any typographical or grammatical errors should be corrected at revision, so please note any specific errors here.

Reviewer #1: Yes

**********

6. Review Comments to the Author

Please use the space provided to explain your answers to the questions above. You may also include additional comments for the author, including concerns about dual publication, research ethics, or publication ethics. (Please upload your review as an attachment if it exceeds 20,000 characters)

Reviewer #1: (No Response)

**********

7. PLOS authors have the option to publish the peer review history of their article (what does this mean?). If published, this will include your full peer review and any attached files.

If you choose “no”, your identity will remain anonymous but your review may still be made public.

**Do you want your identity to be public for this peer review?** For information about this choice, including consent withdrawal, please see our Privacy Policy.

Reviewer #1: No

# Editor report: Acceptance letter

**DOI:** 10.1371/journal.pone.0227220.r004

**Authors:** Amitava Mukherjee

18 Dec 2019

PONE-D-19-29053R1 

Essential Oil-Incorporated Carbon Nanotubes Filters for Bacterial Removal and Inactivation 

Dear Dr. Yang:

I am pleased to inform you that your manuscript has been deemed suitable for publication in PLOS ONE. Congratulations! Your manuscript is now with our production department. 

If your institution or institutions have a press office, please notify them about your upcoming paper at this point, to enable them to help maximize its impact. If they will be preparing press materials for this manuscript, please inform our press team within the next 48 hours. Your manuscript will remain under strict press embargo until 2 pm Eastern Time on the date of publication. For more information please contact onepress@plos.org.

For any other questions or concerns, please email plosone@plos.org. 

Thank you for submitting your work to PLOS ONE.

With kind regards,

PLOS ONE Editorial Office Staff

on behalf of

Professor Dr. Amitava Mukherjee 

Academic Editor

PLOS ONE
